# Design Optimization for the Measurement Accuracy Improvement of a Large Range Nanopositioning Stage

**DOI:** 10.3390/s16010084

**Published:** 2016-01-11

**Authors:** Marta Torralba, José Antonio Yagüe-Fabra, José Antonio Albajez, Juan José Aguilar

**Affiliations:** 1 Centro Universitario de la Defensa Zaragoza, Academia General Militar, Carretera Huesca s/n, Zaragoza 50090, Spain; 2 Instituto de Investigación en Ingeniería de Aragón, Universidad de Zaragoza, María de Luna 3, Zaragoza 50018, Spain; jyague@unizar.es (J.A.Y.-F.); jalbajez@unizar.es (J.A.A.); jaguilar@unizar.es (J.J.A.)

**Keywords:** error budget, 2D-platform, nanopositioning, rigid body behavior, FEA static analysis, atomic force microscopy

## Abstract

Both an accurate machine design and an adequate metrology loop definition are critical factors when precision positioning represents a key issue for the final system performance. This article discusses the error budget methodology as an advantageous technique to improve the measurement accuracy of a 2D-long range stage during its design phase. The nanopositioning platform NanoPla is here presented. Its specifications, e.g., XY-travel range of 50 mm × 50 mm and sub-micrometric accuracy; and some novel designed solutions, e.g., a three-layer and two-stage architecture are described. Once defined the prototype, an error analysis is performed to propose improvement design features. Then, the metrology loop of the system is mathematically modelled to define the propagation of the different sources. Several simplifications and design hypothesis are justified and validated, including the assumption of rigid body behavior, which is demonstrated after a finite element analysis verification. The different error sources and their estimated contributions are enumerated in order to conclude with the final error values obtained from the error budget. The measurement deviations obtained demonstrate the important influence of the working environmental conditions, the flatness error of the plane mirror reflectors and the accurate manufacture and assembly of the components forming the metrological loop. Thus, a temperature control of ±0.1 °C results in an acceptable maximum positioning error for the developed NanoPla stage, *i.e.*, 41 nm, 36 nm and 48 nm in X-, Y- and Z-axis, respectively.

## 1. Introduction

In the last decades, an important line of research in the multidisciplinary nanotechnology field has focused on the development of new accurate positioning devices and technologies [1,2]. These systems are a common element in all manufacturing, manipulation or sample characterization operations, whose performance is a key issue for their final application. The metrological challenge here underlies in the achievement of accuracy, repeatability and stability at submicrometer and nanometer scale. Nonetheless, not only these demanding requirements, but also longer travel ranges have to be considered [3,4]. The common range of devices for nanotechnology issues should be amplified to increase the number of potential applications [5,6].

In order to obtain a new large range stage for nanopositioning, it is necessary to carefully design the system. This phase includes the definition of the conceptual design and the detailed prototype [7]. The conceptual design satisfies all the established requirements. On the other hand, the detailed model of the system is the result of its evaluation and optimization after analyzing each subsystem. That supposes to consider not only all precision engineering principles and applied solutions found in similar stages [8,9], but also also simulations to optimize mechanical and dynamic behavior. The aim is to obtain a predictive design [10]. According to this, error reduction and compensation techniques are focused on the accuracy of the system by determining the number of error influences, quantifying them and latterly, minimizing their effect in the measurement. Calibration is the methodology applied after a machine is set-up, to assure traceability during positioning. On the contrary, error budget is used as a deterministic tool in early development phases to identify, predict and minimize (or even eliminate) measurement error contributions [11,12]. Although some of the error sources could be reduced after applying specific design principles, in some cases, as the one here presented, applying such a technique is necessary in order to meet the accuracy and precision requirements. As a first step, all the physical error causes affecting the system are identified and quantified. Secondly, the mathematical measurement model of the machine establishes the cause-and-effect relationships between them. Hence, some solutions and strategies may be established to improve the measurement accuracy and to optimize the positioning task, according to the demonstrated dominant disturbances and their propagation around the close loop control system.

The goal of the error budget is to estimate the maximum measurement error vector of the developed system before it is manufactured and assembled. Therefore, the methodology begins with the definition of the mathematical measurement model in order to determine the difference between the actual displacement and the desired motion of the sample, tool or probe. Some examples of these may be found in the literature, like in [13,14], where similar kinematic error models are presented for two nanopositioning systems developed at the University of North Carolina at Charlotte, estimating the volumetric standard uncertainty. The value obtained depends in all the cases on the contribution of the different error sources, which could be classified into three groups as shown in [15,16]: *i.e.*, instrumental, alignment and environmental errors. By applying the error budget technique, these particular error sources and their way of propagation around the measurement loop can be known, making possible to propose design modifications in order to obtain the best technical and economical solutions [17].

In this article, an error budget analysis is presented to improve the measurement accuracy of a novel nanopositioning platform (NanoPla) during its design phase. This novel two-dimensional large range stage (50 mm × 50 mm) has been developed to provide nanometer resolution for the displacement feedback and sub-micrometer position uncertainty. The main design novelty of the system is based on its three-layer and two-stage architecture. This scheme supposes compactness, which improves the metrological performance. Once defined the initial design, the metrology loop of the system is mathematically modelled. This expression of the error vector in terms of positioning defines the propagation of the different sources. Then, the study continues with the different error sources definition and estimation. The final measurement error is evaluated along the working range of the system in order to optimize the conceptual design of the platform [18]. Particular design improvements can then be proposed, after the differentiation and quantification of the error sources, in view of their contribution to the final vector result.

## 2. Nanopositioning Stage: Design Overview

The NanoPla project is focused on the development of a 2D-long range stage capable of providing accurate positioning for different nanotechnology tasks: e.g., sample characterization, nanoprobe calibration, nanomanufacturing processes, *etc.* The performance requirements considered desirable for a broad variety of applications using this system as a common positioning stage are the following:

- XY-travel range = 50 × 50 mm^2^
- XY-resolution = 10 nm
- XY-positioning error = 25 nm
- Scanning speed > 2 mm/s

The first integrated tool to be used with the NanoPla stage will be an AFM [19]. This system offers high vertical, as well as, lateral system resolution for nanostructure evaluation. It will be used to characterize larger specimens without cutting samples and maintaining a global reference over the long travel range. However, this first NanoPla prototype also integrates an auxiliary nanostage for the scanning task to assure the right interaction between sample and probe. The long range motion of the AFM is carried out and measured by actuators and sensors, respectively. Four Halbach linear motors are used to create dual forces (horizontal and vertical components) between the stators and magnetic arrays. The horizontal components provide a frictionless motion of the moving part of the system, which is in levitation thanks to the three vacuum preloaded air bearings, while the vertical components of the forces help to this levitation. Six are the Degrees of Freedom (DoFs) to be evaluated by the measurement system: three translations (XYZ) and three rotations R_x_R_y_R_z_ around X-, Y- and Z-axis, respectively. The adopted solution to measure the XYR_z_-DoFs is a laser interferometer sensor system using plane mirrors as reflectors. Capacitive probes are used for the out-of-plane motions measurement: ZR_x_R_y_-DoFs.

The final design of the stage is based on an extensive review of the state-of-art concerning similar systems and technologies. It applies precision engineering principles and desirable features found in the literature [20,21,22]. The final architecture of the whole system is defined by a three-layer and a two-stage scheme, obtaining a compact and symmetrical design. Additionally, commercial components have been used when possible. Finally, the system has been validated by finite element simulation, analyzing deformations and vibration modes of the complete stage and individual parts.

### 2.1. Three-Layer Scheme

**Figure 1:**
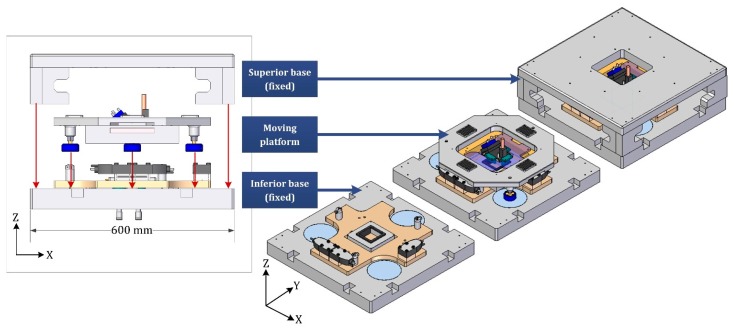
Three-layer NanoPla novel architecture with related parts. (**Left**) Exploded front view of the prototype; (**Right**) 3D-model schemes including different parts.

The main characteristic of our stage is the novel arrangement based on compactness for a long travel range in nanopositioning applications. The three-layer solution applied, comprising a fixed superior base, a moving central platform and a fixed inferior base, is shown in Figure 1. As illustrated, the moving platform is installed between the two fixed bases. The final size of the whole machine is approx. 600 mm × 600 mm in the footprint, and approx. 200 mm in height. This design assures the characterization of big samples, an AFM motion capability over 50 × 50 mm^2^ and no collisions between elements. In addition, the use of three different layers allows placing closer to each other the linear motors and reduces the total footprint.

**Figure 2:**
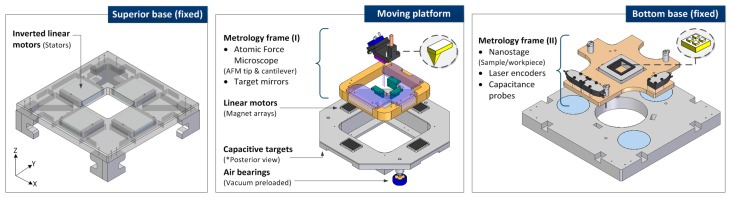
Detailed components view of the NanoPla three-layer main parts. (**Left**) Superior base; (**Middle**) Moving platform; (**Right**) Bottom base.

The detailed views of each layer are illustrated in Figure 2. The structural parts are made of aluminum alloy 7075-T6, due to its excellent technical characteristics: low density, corrosion resistance, good heat conductivity, easy and economic processing. The superior part integrates the bulky stators in an inverted position. This innovative configuration allows the relative long travel motion and reduces the whole stage size. The moving platform has a frictionless displacement due to the actuators and the levitation system that supports this part over the bottom base. Regarding the metrological loop, two metrology frames made of Zerodur are integrated to locate the sensor systems. The metrology frame (I) of the moving platform supports the AFM (tool or probe) and the plane mirrors. The metrology frame (II) of the fixed bottom base incorporates the nanostage (sample), the three laser interferometer heads for X-, Y-axis and yaw measurements, and the three capacitance probes to quantify small deviations in Z-direction, pitch and roll orientations.

### 2.2. Two-Stage Motion Strategy

The two-stage motion strategy is based on the use of a complementary fine motion system for the sample scanning. Then, the whole range is characterized by the measurement of little areas. In other words, the platform here designed displaces the tool in the XY-plane 50 mm × 50 mm, while the scanning task is carried out by a commercial nanopositioning stage. Hence, the AFM (long range stage) is stationary during the topographic profile mapping, and the sample is moved with the short range XYZ-nanopositioner (100 μm × 100 μm × 10 μm).

### 2.3. FEA Validation

The described NanoPla 3D-model has been verified and optimized by Finite Element Analysis (FEA). Structural static and modal analyses have been carried out by ANSYS^®^ software with two main goals. First, the geometries of each part have been determined, in view of their specific mechanical requirements (stiffness, lightness, *etc.*). The second approach is focused on the assumption of rigid body behavior as one of the hypothesis that the error budget works with. This additional static analysis is justified because of the high NanoPla moving mass and the demanding positioning requirements. For this reason, these analyses have been carried out at a sub-micrometric scale.

**Figure 3:**
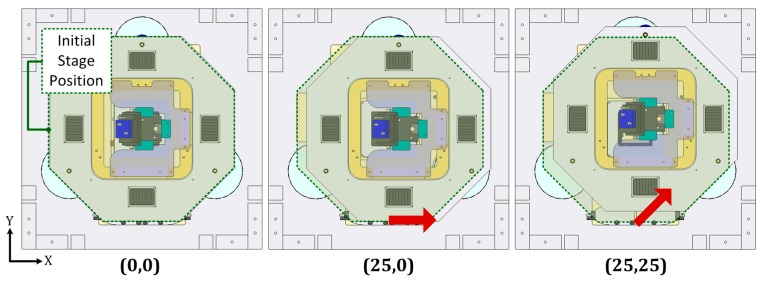
Considered NanoPla representative 2D-motions along the long travel range. (**Left**) Initial stage position; (**Middle**) X-displacement of 25 mm; (**Right**) XY-displacement of (25,25) mm.

**Figure 4:**
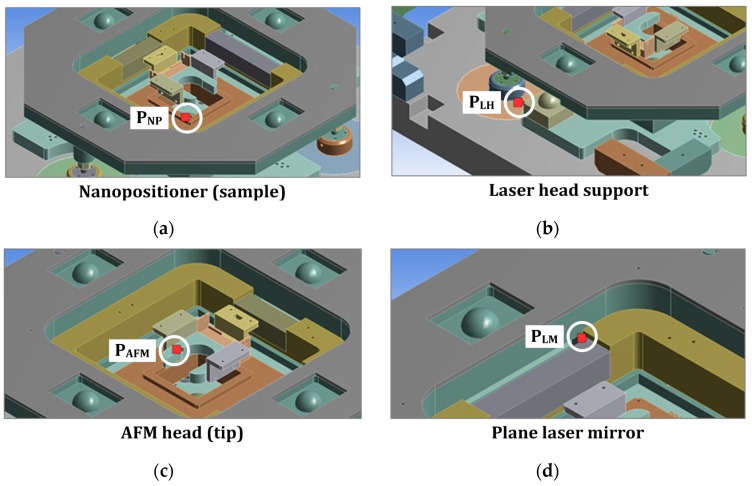
Analyzed points of the metrology loop of the detailed FEA simulations. (**a**) P_NP_, nanopositioner; (**b**) P_LH_, laser head; (**c**) P_AFM_, AFM head; (**d**) P_LM_, laser mirror.

An additional convergence test has been developed to define the element size of the mesh that provides sensitivity in the deformation results at nanometer scale. Afterwards, four significant points of the metrology loop have been selected to estimate the relative position change when displacing the stage to the extreme positions of the long travel range. These positions over the long travel range are described as (L_x_,L_y_) and the three most representative are shown in Figure 3: (0,0); (25,0) and (25,25), respectively. The four (x,y,z) points are characterized for being part of the metrology loop. As illustrated in Figure 4, they are the following:

- Metrology frame (I): P_AFM_, a point of the tool (AFM mounting piece closest to the AFM tip) and P_LM_, the edge of the longest plane laser mirror.
- Metrology frame (II): P_NP_, a point in the nanopositioner (sample) and P_LH_, the most critical point of the laser head support (higher deformation).

The mesh convergence has been analyzed by registering the XYZ-deformations of the four points, when the used tetrahedron elements size was progressively decreasing. The final size of the applied tetrahedrons obtained was 2 mm, which provides deformation changes with nanometer sensitivity. The relative displacements of the four considered points along the long range positions are presented in Table 1, considering the cited element size in the simulations. 

As a result, these differences are around zero in several cases and the highest value is approximately 13 nm. Nevertheless, the trend is characterized by achieving almost equivalent relative displacements in all components. In conclusion, the global change or deviation of the metrological loop part analyzed is almost negligible and also equivalent in all the positions. Thereby, a solid rigid body behavior can be assumed, despite the significant moving mass and the required operation features at nanometer scale of the final system application.

**Table 1:** Relative displacements of the interesting points (detailed FEA).

Analyzed Points	Considered 2D-Long Range Displacements (mm)			Relative Point Displacements (nm)		
**P_i_**	**(L_x_,L_y_)_i_**	**→**	**(L_x_,L_y_)_f_**	**ΔP_i,x_**	**ΔP_i,y_**	**ΔP_i,z_**
**P_AFM_**	(0,0)	→	(25,0)	−0.17	−12.72	4.30
	(0,0)	→	(25,25)	−6.75	−7.17	7.00
	(25,0)	→	(25,25)	−6.58	5.54	2.70
**P_LM_**	(0,0)	→	(25,0)	0.02	−12.79	4.18
	(0,0)	→	(25,25)	−6.63	−7.31	6.75
	(25,0)	→	(25,25)	−6.65	5.48	2.57
**P_LH_**	(0,0)	→	(25,0)	0.00	0.01	0.00
	(0,0)	→	(25,25)	0.01	0.01	0.00
	(25,0)	→	(25,25)	0.01	0.00	0.00
**P_NP_**	(0,0)	→	(25,0)	0.06	−12.86	4.30
	(0,0)	→	(25,25)	0.02	−7.33	6.90
	(25,0)	→	(25,25)	−0.04	5.53	2.60

## 3. Error Vector for the NanoPla

The error budget is a well-established and advantageous technique to optimize the performance of a system during its design phase. Its utility is based on the study and quantification of the different error sources that affect the metrology loop. For this reason, the methodology begins with the definition of the mathematical measurement model. This model determines the difference between the actual displacement and the desired motion of the sample, tool or probe. Thus, the error vector for the NanoPla is presented in this Section 3. Considered hypotheses are justified and specific applied simplifications are also described.

### 3.1. Mathematical Error Model

The developed 2D-moving platform and its sensor configuration measure the 6-DoFs: *i.e.*, three translations and three rotations. However, its actuation and control scheme is different from those in machine tools or CMMs [23]. In these classic models, error analyses are based on the superposition of linear movements. Kinematic chains establish the relation and propagation between these errors that are studied independently. On the contrary, the NanoPla’s linear motors provide a two-dimensional actuation. Thus, some errors in the horizontal plane will be related to each other and considered in the geometric model.

**Figure 5:**
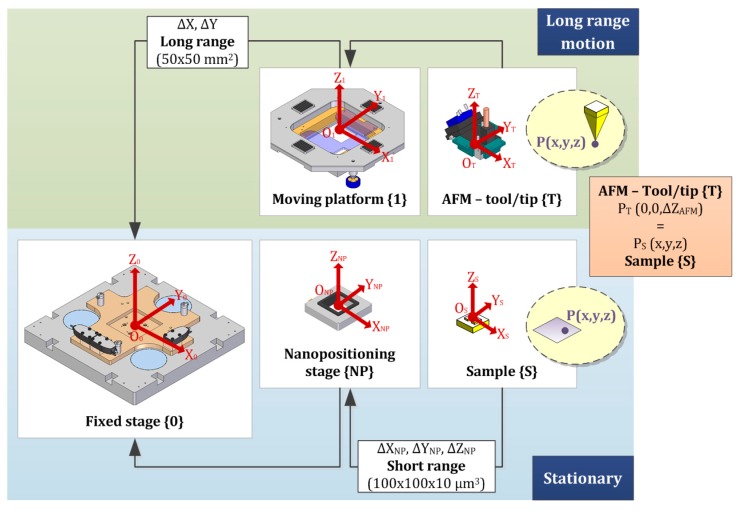
Schematization of the complete measurement loop of the NanoPla stage.

In addition to the geometric relationships in the XY-plane, the two-stage motion strategy should also be mathematically considered. The global scheme of the measurement loop is shown in Figure 5. The long range moving platform, coordinate system {1}, provides the 50 mm × 50 mm displacement to the AFM tip {T}. The base is kept fixed {0}, like the nanopositioning stage placed over it {NP}. This short range nanopositioning device moves the sample {S} during the scanning task in the smaller volume of 100 × 100 × 10 μm^3^. Therefore, both main parts are linked by the stationary base or fixed stage {0}. The two chains associate points of the sample and tip location. The point of the AFM tip P_T_ (x,y,z) should ideally meet the scanned point of the sample P_S_ (x,y,z) according to the same common reference of coordinate system {0}. In other case, differences between two locations will be consequence of deviations in the measurement task. The aim of this part of the work is to conclude with the mathematical expression that relates P_T_ and P_S_ with transformations between coordinate systems: translations of the arbitrary origins and orientation changes or rotations. The five considered systems define the following relations (see Figure 5):

- Superior chain:
  (1) $$P_{1}=R_{1}^{T}P_{T}+T_{1}^{T}$$
  (2) $$P_{0}=R_{0}^{1}P_{1}+T_{0}^{1}$$
- Inferior chain:
  (3) $$P_{NP}=R_{NP}^{S}P_{S}+T_{NP}^{S}$$
  (4) $$P_{0}=R_{0}^{NP}P_{NP}+T_{0}^{NP}$$

P_i_ is the analyzed point expressed according to (i) system; R_ij_ is the rotation matrix from (i) to (j) systems; and T_ij_ is the translation vector between O_i_ and O_j_ origins.

In view of the common reference {0}, P_T_ and P_S_ are related as seen in the continuity Equation (5):

(5) $$P_{S}={\left[R_{NP}^{S}\right]}^{-1}{\left[R_{0}^{NP}\right]}^{-1}R_{0}^{1}R_{1}^{T}P_{T}+{\left[R_{NP}^{S}\right]}^{-1}{\left[R_{0}^{NP}\right]}^{-1}R_{0}^{1}T_{1}^{T}+{\left[R_{NP}^{S}\right]}^{-1}{\left[R_{0}^{NP}\right]}^{-1}T_{0}^{1}-{\left[R_{NP}^{S}\right]}^{-1}T_{NP}^{S}-{\left[R_{NP}^{S}\right]}^{-1}{\left[R_{0}^{NP}\right]}^{-1}T_{0}^{NP}$$

### 3.2. Considered Hypotheses

Three are the main considered hypothesis in this error budget analysis: *i.e.*, two-dimensional plane motion assumption; rigid body behavior and independent errors; and small angular errors.

- *Two-dimensional plane motion*: In the NanoPla stage, the XY-long range displacement supposes only the movement of one structure in the horizontal plane, without linear motions superposition. Hence, there will be related geometric errors contributions, such as the misalignment between the two target mirrors, one per axis.
- *Rigid body behavior and independent errors*: As previously presented, the rigid body behavior hypothesis turns satisfactory after the FEA simulations. The total displacement of four strategic points has shown a relative non-existent displacement between parts when the moving platform achieves different extreme positions of the long travel range. Thus, under the assumption of rigid body behavior, systematic considered errors in each axis are only dependent on the displacement along that axis.
- *Small angular errors*: Angular errors are assumed to be small angles (<0.05 rad) to simplify the composed rotation matrix in the three axes. This supposition is realistic, according to the analyzed case. Angular deviations during operation of the stage should be at submicrometer scale. However, this matrix is not orthonormal, so that some minor perpendicularity errors are included in the model.

### 3.3. Model Simplifications

Several points are considered to conclude in a simpler mathematical expression of the measurement loop model, *i.e.*, Equation (5), for this initial error contribution study:

- Coordinate transformation {0}–{1} (from the fixed stage to the moving platform):

This transformation characterizes the long range motion. The laser interferometer and the capacitive sensors readouts determine the movement of the moving platform on the 6-DoF. Therefore, they are related to the translation and rotation transformations of coordinate systems {0} and {1}. For the ideal case (*i.e.*, no errors in the 2D-motions), sensor signal responses can be simplified. The X- and Y-parameters are the desired displacements in the horizontal plane. This pure motion along the XY-plane results in a zero Z-displacement. In addition, the difference between the origins is assumed non-existent. These fixed parameters will be known and optimized once the platform will be operating. The uncertainty of their measurement is not considered in the error budget. On the contrary, different errors may be included in the terms of the translation and rotation matrices, due to the deviations during the measurement of the positioning. For the NanoPla case, they are listed in Table 2.

**Table 2:** Considered errors related to the {0}-{1} transformation.

Geometric Error	Description	Parameter
**Motion errors** (3)	Error motion in X-axis	**δ_x_**
	Error motion in Y-axis	**δ_y_**
	Error motion in Z-axis	**δ_z_**
**Rotation errors** (3)	Rotation of {1} with respect to {0} about X_0_ (pitch)	**ε_x_**
	Rotation of {1} with respect to {0} about Y_0_ (roll)	**ε_y_**
	Rotation of {1} with respect to {0} about Z_0_ (yaw)	**ε_z_**
**Orthogonality errors** (3)	Angle between X_0_ and X_1_ projection onto X_0_Y_0_ plane	**α_xy_**
	Angle between Y_0_ and Y_1_ projection onto Y_0_Z_0_ plane	**α_yz_**
	Angle between X_0_ and X_1_ projection onto X_0_Z_0_ plane	**α_zx_**

If distances are considered in [mm], angles in [rad], and if the origin of the travel range is coincident with the center of the 3D-stage model, the translation vector $T_{0}^{1}$ and rotation matrix $R_{0}^{1}$ are as follows:

(6) $$T_{0}^{1}=\left[\begin{array}{c} L_{x}+\delta_{x}\\ L_{y}+\delta_{y}-\alpha_{xy}L_{x}\\ \delta_{z}-\alpha_{yz}L_{y}+\alpha_{zx}L_{x} \end{array}\right]$$

(7) $$R_{0}^{1}=\left[\begin{array}{ccc} 1 & -\varepsilon_{z} & \varepsilon_{y}\\ \varepsilon_{z} & 1 & -\varepsilon_{x}\\ -\varepsilon_{y} & \varepsilon_{x} & 1 \end{array}\right]$$

- Coordinate transformation {1}–{T} (from the moving platform to the AFM):

The transformation between the moving platform {1} and the tool {T} is mainly characterized by the location of the AFM tip origin with respect to the coordinate system {1}: $T_{1}^{T}$ and $R_{1}^{T}$. As before, origins are considered coincident and also rotations are negligible. However, considering the $T_{1}^{T}$ translational vector, the cited assumption could affect the model. In view of Equation (5), this term is multiplied by the rotation matrix $R_{1}^{T}$, which depends on the errors of Table 2. If $R_{0}^{1}$ is not equal zero, this assumption would be incorrect, according to the error propagation. It has been verified that measurement errors are less than 3 nm for displacements between origins of around five millimeters. Therefore, its error contribution would be negligible, so that the simplification is acceptable.

- Coordinate transformation {0}–{NP} (from the fixed stage to the nanopositioning stage):

In this case, for both fixed systems {0} and {NP}, it turns easy to consider coincident origins ($T_{0}^{NP}$ equals zero) and negligible rotations ($R_{0}^{NP}$ equals the identity matrix). After the assembly, these parts will maintain their relative position.

- Coordinate transformation {NP}–{S} (from the nanopositioning stage to the sample):

The transformation that involves coordinate systems {NP} and {S} is affected by the scanning motion of the sample (located over the nanopositioning stage). Hence, the introduced errors are a function of the nanopositioner architecture and performance. As before, {O_0_} and {O_NP_} origins are assumed coincident and rotations are removed. Nevertheless, in this transformation the initial hypothesis of small angles makes no sense. The sample could have different orientations or particular geometry. Its reference system could change from one workpiece to another, which is out of this error analysis. The datasheet information of a commercial fine movement stage (NPXY100Z10A of nPoint, Inc. (Middleton, WI, USA)) only includes linearity errors for all the three Cartesian axes of motion. As it is shown in Equation (8), their contribution affects the translation vector $T_{NP}^{S}$ and includes the displacement given by the nanostage:

(8) $$T_{NP}^{S}=\left[\begin{array}{c} \Delta X_{NP}+l_{error,x}\Delta X_{NP}\\ \Delta Y_{NP}+l_{error,y}\Delta Y_{NP}\\ \Delta Z_{NP}+l_{error,z}\Delta Z_{NP} \end{array}\right]$$

where ΔX_NP_, ΔY_NP_ and ΔZ_NP_ are the traversed distance in the scanning motion in [mm] and l_error,I_, being i=X,Y,Z, the non-linearity error of the commercial nanopositioner.

- Final balance with errors

After simplifications, the considered Equation (5) for the error budget results in Equation (9) as follows:

(9) $$P_{S}=R_{0}^{1}P_{T}+T_{0}^{1}-T_{NP}^{S}$$

The input data of the P_T_ vector is the Z-positioning of the AFM tip with respect to the {T} coordinate system. Constant cantilever deflection with vertical fine motions of the sample supposes to keep stationary the Z-position of the tip during the scanning. Hence, this vector can be assumed equal to zero. Vertical displacements and AFM errors are supposed negligible, so that P_T_ has no influence in this analysis. The translation vector for the coordinate transformation 0–1 is a function of L_x_ and L_y_, as shown in Equation (6). Both input parameters are ±25 mm for the long travel working range. In addition, $T_{NP}^{S}$ is established according to the volume of the nanopositioning stage. If the origin is placed on the central point of the range, $\Delta X_{NP}=\Delta Y_{NP}$ and $\Delta Z_{NP}$ could achieve ±50 μm and ±5 μm, respectively. Thus, the final vector that defines the difference between the AFM tip and the measured point of the sample is as seen in the continuity Equation (10), where $\Delta P_{S}(\Delta P_{S,x},\Delta P_{S,y},\Delta P_{S,z})$ is specified:

(10) $$\Delta P_{S}=\left[\begin{array}{c} \Delta P_{S,x}\\ \Delta P_{S,y}\\ \Delta P_{S,z} \end{array}\right]=\left[\begin{array}{c} \delta_{x}\\ \delta_{y}-\alpha_{xy}L_{x}\\ \delta_{z}-\alpha_{yz}L_{y}+\alpha_{zx}L_{x} \end{array}\right]+\left[\begin{array}{c} l_{error,x}\Delta X_{NP}\\ l_{error,y}\Delta Y_{NP}\\ l_{error,z}\Delta Z_{NP} \end{array}\right]$$

## 4. Error Budget Analysis

Once the error vector of the stage is specified, we can proceed with the enumeration of the error sources and estimation of their corresponding contribution. According to the Guide to the expression of Uncertainty in Measurement [24] and ISO 14253-2:2011 [25], if these errors are expressed in probabilistic terms and the measurement model is perfectly defined, an uncertainty analysis can be obtained (as in the work presented in [26]). In other words, the uncertainty analysis assumes to know the detailed measurement model and different parameters that are here simplified, in view of this initial design phase. Hence, the propagation of uncertainty based on the GUM and ISO methodologies will be evaluated in future works, after considering all parameters of the model and after determining the exact mathematical expression of the measurement.

Focusing on the different error sources, they can be classified into three groups: instrumental, alignment and environmental errors. Their consideration and contribution to the final model is here justified. Other additional error sources, such as vibrations and deformations (mechanical behavior) are not considered here since their influence is minor in this case and since their evaluation would need of some previous mechanical experiments of the stage, not possible at this design phase [26]. For similar reasons stability is also omitted. To conclude, this part of the article concludes with the error budget results, previously to the conclusions and final discussion, in order to contribute to the design optimization of the 2D-moving platform.

### 4.1. NanoPla Error Sources

The different error influences that affect the nanopositioning task of our prototype are here classified into three groups: instrumental, alignment and environmental sources. Instrumental sources include all the measurement discrepancies due to the used sensor system (*i.e.*, resolution, wavelength stability, *etc.*). Alignment issues are related to sine and cosine errors, and parallelism or orthogonality between elements. Form imperfections of reflectors have also some influence, because of their non-uniform surface (global and local flatness). They can be included in this second group. Environmental changes affect in two ways: variations in the air refractive index of air (laser wavelength instabilities) and thermal expansion of components, holders and sensors. Thereby, this point describes and estimates all the included error contributions of the cited groups. Their final contributions are summarized in Table 3 presented at the end of this Section 4.

**Table 3:** NanoPla error budget: summary of the different error sources and their contribution.

Error Source	Description	Error Contribution (nm)	Influence
**Instrumental Plane mirror laser interferometers**	Wavelength instability (Laser frequency stability)	<±50 ppb (1–8 h) → ±1.25 nm	δ_x,_ δ_y_
	Sensor resolution	≈1.6 nm	δ_x,_ δ_y_
		2.91 × 10^−8^ rad	ε_z_
	Beam mixing (Nonlinear optics, polarization, *etc.*)	<±2 nm below 50 mm/s with >70% signal strength	δ_x,_ δ_y_
**Instrumental Capacitive sensors**	Sensor resolution	10 nm	δ_z_
		3.08 × 10^−8^ rad	ε_x_, ε_y_
**Instrumental Nanopositioning stage**	Linearity error	X&Y = 0.05%Z = 0.5%	l_error,x_ = l_error,y_l_error,z_
**Alignment**	Laser beam & plane mirror alignment	X-mirror alignment: β_x_ = 8.02 × 10^−6^ rad	δ_x,_ δ_y_
		Incident beam alignment:	
		θ_x_ = θ_y1_ = θ_y2_ = 6.06 × 10^−5^ rad	
		$\delta_{x}=f(L_{x})$ → Equation (11)	
		$\delta_{y}=f(L_{y1},L_{y2})$ → Equation (12)	
	Orthogonality between plane mirrors (CMM)	±1.09 × 10^−6^ rad	α_xy_
	Orthogonality between plane mirrors-capacitive targets (CMM)	±1.14 × 10^−6^ rad±1.31 × 10^−6^ rad	α_zx_α_yz_
	Parallelism between capacitive probe and target	Established 25 nm	δ_z_
	Form errors	<λ/10 per 100 mm (λ = 633 nm) → 31.5 nm	δ_x,_ δ_y_
	Plane mirror global flatness	<λ/10 per 100 mm (λ = 633 nm) → 5.72·10^−7^ rad	ε_z_
**Environmental influence**	Real time quadrature compensation laser system	Accuracy ±1 ppm → ±0.25 nm	δ_x,_ δ_y_
		(∆T = ±0.1 °C); ±2.5 nm (±1 °C)	
	Thermal expansion: capacitive targets (thermal loop)	±10.4 nm (∆T= ± 0.1 °C);	δ_z_
		±104 nm (±1 °C)	
	Thermal expansion: capacitive probes (own material and coupling)	±8.6 nm (∆T = ±0.1 °C);	δ_z_
		±85.8 nm (±1 °C)	

#### 4.1.1. Instrumental Errors

Instrumental errors are directly related to the devices that comprise the metrological loop: the sensor systems, the AFM and the commercial nanopositioning stage.

The laser system introduces δ_x_, δ_y_ and ε_z_ geometric deviations when measuring the three XYR_z_-DoFs. Particularly, the commercial instruments of Renishaw plc. (Wotton-under-Edge, UK) assume the following contributions: wavelength instability of the RLU10 laser unit and the ±25 mm required displacement affects ±1.25 nm (1–8 h) in the motion error. Due to the REE interpolator, resolution error is approx. 1.6 nm (δ_x_ and δ_y_) and 29.1 nrad (ε_z_). Beam mixing or spurious beams lead to a non-linearity error less than ±2 nm in the RLD10–90° detector head. Finally, because of the interferometer architecture, there is no dead-path error.

The Lion Precision (St Paul, MN, USA) capacitive sensors (C5-E) used for evaluating out-of-plane deviations (ZR_x_R_y_) add instrumental errors as a consequence of their resolution. Considering their standard value and the NanoPla configuration (sensor location), the resolution error of the capacitive probes is 10 nm (δ_z_) and 30.8 nrad (ε_x_ and ε_y_). Linearity error specification of 0.02% supposes a negligible contribution.

The other system that comprises the metrology loop is the nanostage. Noise during operation is less than 1 nm, so that it results insignificant. The main error source is a consequence of the lack of linearity in the 100 × 100 × 10 μm^3^ working range. Linearity errors of the nanopositioning stage included in the datasheet are 0.05% for X- and Y-axis, and 0.5% for Z-motions.

#### 4.1.2. Alignment Errors

**Figure 6:**
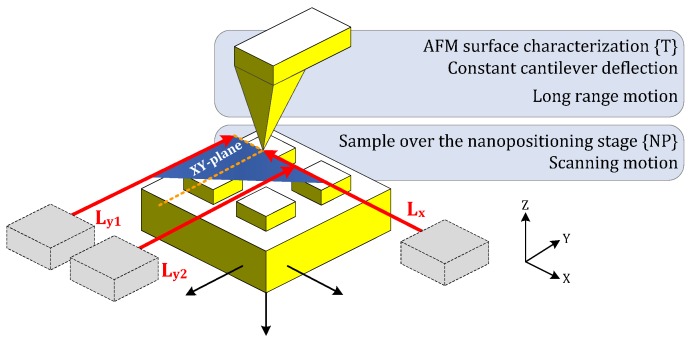
Laser beams and AFM tip alignment: Fulfilled 2D-Abbe principle.

Alignment errors are a consequence of two aspects: the own location of elements by design (*i.e.*, Abbe error) and the uncertainty in the parts positioning with metrological function during the assembly (for example, sensor probes and targets).Abbe error is minimized by design. As shown in the scheme of Figure 6, a right alignment exists between the laser beams and the AFM tip. The AFM tip is coincident with the center of the reference system. The laser support and the adopted architecture allow the right vertical alignment. That concludes in an inexistent Abbe error in the horizontal plane. However, first and second order errors could appear. The performance of the nanopositioning stage during the scanning concerns the accurate positioning. In spite of that, the tip is almost fixed during the surface measurement, according to the AFM control strategy of keeping constant the cantilever deflection. Hence, these geometric errors should be negligible.

**Figure 7:**
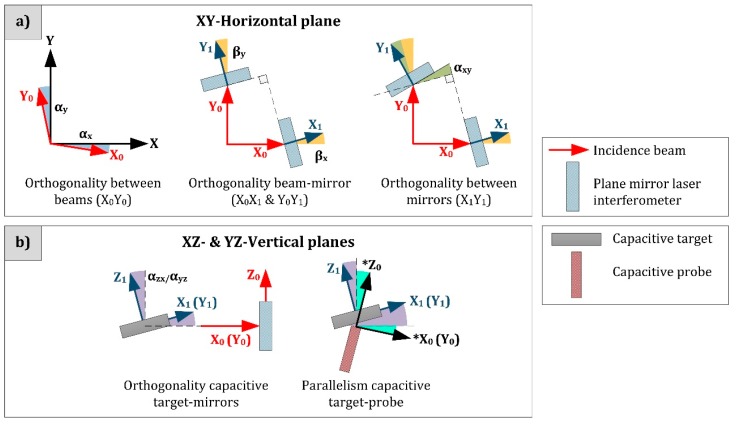
Existing misalignments in (**a**) XY-Horizontal Plane and (**b**) XZ- and YZ-Vertical planes, regarding the sensor scheme of plane mirror laser interferometers and capacitive sensors.

The other misalignment errors appear due to the impossibility of a perfect components assembly. The reference coordinate systems defined by the sensor arrangement will present discrepancies in comparison to the ideal design: inexact alignment in each axis and consequent lack of squareness between all axes. These differences could appear in three planes considering the laser beams (XY-Horizontal plane, Figure 7a), the plane mirror reflector and the capacitive target and probe (XZ- and YZ-Vertical planes, Figure 7b). Specific causes and computation of the errors are described in the following paragraphs.

**Figure 8:**
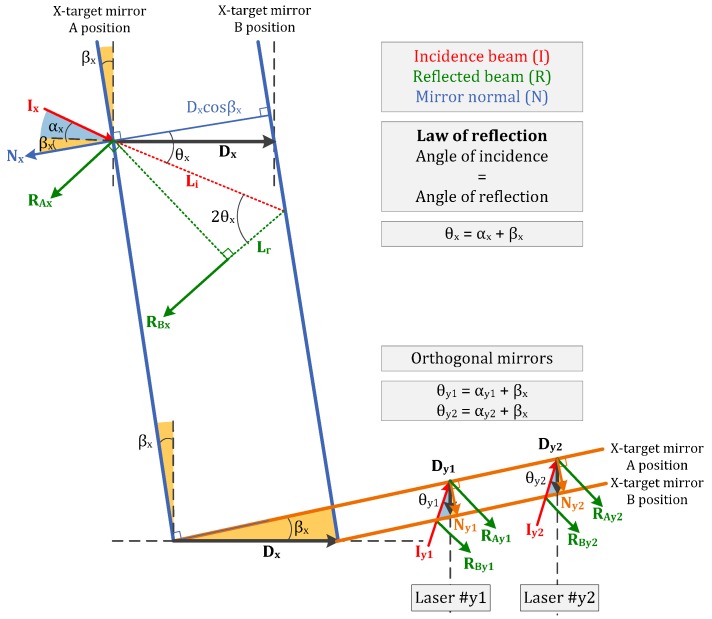
Considered angular deviations due to the laser beams and plane mirrors orthogonality errors in a 2D-motion stage scheme.

Studying first the XY-plane errors, the squareness between laser beams and the orthogonality of each beam and the plane mirror surface are geometrically detailed in Figure 8. The α-angle represents the lack of parallelism of the incident laser beam and the ideal reference axis of motion. The β-angle is the lack of squareness, considering the mirror surface and the ideal reference axis of motion.

If we consider both influences, the following motion errors are obtained, regarding the schematization of Figure 8.

(11) $$\delta_{x}=\left(\frac{1}{\cos\beta_{x}\cos\theta_{x}}-1\right)L_{x}$$

(12) $$\delta_{y}=\frac{1}{2}\left(\frac{1}{\cos\beta_{x}\cos\theta_{y1}}-1\right)L_{y1}+\frac{1}{2}\left(\frac{1}{\cos\beta_{x}\cos\theta_{y2}}-1\right)L_{y2}$$

The question here is how to determine the error sources that affect the input values of Equations (11) and (12): $\beta_{x},\theta_{x},\theta_{y1},\theta_{y2}$ ($L_{x},L_{y1},L_{y2}$are laser readouts). They depend on technical features of the laser system and manufacturing and assembly issues. The β_x_-angle is a function of the uncertainty during the installation and adjustment of the plane mirror according to the reference axis. In the worst case, measuring the shortest mirror (80 mm length) with a particular Coordinate Measuring Machine (CMM), β_x_ has a value of 8.02 × 10^−6^ rad. Regarding the Renishaw RLD10-90° detector head, the beam alignment tolerance for the plane mirror is ±25 arc seconds (1 m axis and applied tolerance to both pitch and yaw during operation). That is the value for $2\theta_{x}$, so that $\theta_{x}=\theta_{y1}=\theta_{y2}=$ 6.06 × 10^−5^ rad. The variable that has not been included in the scheme of Figure 8 is the squareness between both plane mirrors (α_xy_). It will also depend on manufacturing and assembly issues. If it is evaluated with the CMM, its angular deviation in the XY-plane supposes a squareness error of α_xy_ = 1.09 × 10^−6^ rad.

Concerning the misalignment errors in the vertical XZ- and YZ-planes, the capacitance sensors influence in the vertical reference system definition. As previously, they relate {0} and {1} coordinate systems (fixed stage and moving platform). The squareness between plane mirrors and capacitance targets is also dependent on the CMM angular deviations. In this case, the results to be included in the error budget are: α_zx_ = ±1.14 × 10^−6^ rad and α_yz_ = ±1.31 × 10^−6^ rad. Due to the difficult task of assuring the parallelism between capacitance probe and target (the small active area limits the characterization of this plane only by measuring the exterior housing), the parallelism error (δ_z_) is established as 25 nm, the half part of an order of magnitude greater than the resolution of the sensor. Other factor that ends in possible changes in the capacitance probe lectures are related to the target shape and surface finish, so that a specific surface quality will be a manufacturing requirement. Nevertheless, the performance of the sensors establishes a small tolerance according to these imperfections. The signal response of the system in terms of capacitance changes is an average, in view of the quality and form of the surface.

A particular problem of alignment is the result of the plane mirror form errors. The non-uniform surface is characterized by a local and global flatness, provided data by the manufacturer of the reflector. The total flatness affects all the useful surface of the mirror and reaches a value less than λ/10 per 100 mm (λ = 633 nm). Despite of the different length of the mirrors, those errors are identical: motion errors of 31.5 nm in X- and Y-axis, and a yaw contribution equal to 5.72 × 10^−7^ rad.

#### 4.1.3. Environmental Influences

Environmental changes produce variations in the air refractive index and thermal expansion effects. The air refractive index is related to the laser wavelength and depends on several parameters: temperature, pressure, humidity and CO_2_ content. Temperature variations that cause material expansion and contraction are studied from ±0.1 °C to ±1 °C, in order to justify the required environmental conditions for the NanoPla system.

Concerning the Renishaw laser system used, the RCU10 unit, it provides a real-time compensation with calibrated sensors, monitoring air refractive index changes as a function of the environmental pressure and temperature. Humidity and CO_2_ content will have a negligible variation under normal working conditions. According to the datasheet of the auxiliary laser unit, the accuracy of the refractive index compensation is ±1 ppm. That means motion errors in the NanoPla XY-positioning of ±2.5 nm.

Dimensional changes of components because of thermal expansion are the other environmental error source. This is a troublesome issue for high precision metrology systems, especially at nanometer scale. In order to avoid relevant discrepancies as a consequence of high thermal changes, the stage and sample should be previously stabilized. Regarding the stage design, it is important to characterize the possible relative changes between elements of the metrology loop: AFM, nanopositioning stage, laser system and capacitive sensors. The commercial scanning probe and its cantilever holder are supposed optimized by design. Dimensional changes because of thermal variations are considered here negligible. The same simplification is established for the nanopositioner. Hence, the importance in the analysis falls on displacements between the main sensor parts: on the one hand, the laser head and the plane mirror; and, on the other hand, the capacitive probe and its target. The hypothesis of a good thermal design for the whole structure is assumed, after application of the principles of symmetry, minimal sensitivity and right management of heat sources. The study is only focused on the metrology frames, which are made of a low expansion coefficient material and isolated with flexure mounts. If a constant thermal expansion coefficient of the material and 1D-expansion are assumed, error contributions can be determined as below.

Renishaw RLE plane mirrors are made of Zerodur, so that dilations are negligible (0.1 ppm/K). Changes in length and height of the mirror do not affect to the measurement. The error is also minimal in width (±2.5 nm in the worst case of ∆T = ±1 °C). Nevertheless, the coupling of the mirrors should be defined to minimize structural deviations by thermal expansion. Considering the design of the laser heads, it is expected that the commercial component barely introduces errors for thermal influences. Their relative position could change due to dimensional modifications of its mounting parts. The supporting plates made of Invar (α_Invar_ = 1.3 μm/mK) could suppose dilations of nanometer scale. In order to avoid them, the union points of the head to these supports are aligned with the laser beam.

Undesired deviations in the XZ- and YZ-vertical planes due to thermal effects are more difficult to avoid by design. Symmetry around the horizontal plane is not possible by design, and dilatations in Z-direction cannot be compensated. Focused on the target of the capacitive sensors, they are located outside the metrology frame, directly coupling to the moving platform. Then, they are not isolated by the flexure arrangement. Their material is steel (α_steel_ = 13 μm/mK) and the thickness has to be enough to allow the machining of the surface. Vertical expansion of the defined element supposes a specific error up 9.1 nm of distance from the probe when ∆T = ±0.1 °C. At the same time and on the contrary, the moving platform may change its vertical position. Made of steel and with the same need of a particular thickness for the surface machining operation, air bearing surfaces will expand approaching the probe (19.5 nm when ∆T = ±0.1 °C). Considering both contributions of the thermal loop, the final estimation of this error source is 10.4 nm if ∆T = ±0.1 °C (up 104 nm for ±1 °C). The capacitive probe is installed in the metrology frame (II). The problem here may be the vertical displacement by thermal changes due to the own probe or its coupling. In addition, the fitting element may introduce some significant errors. Hence, because of the advantages of using Invar, this is the selected material for the shell. If a temperature range of ±0.1 °C is assured, the vertical displacement is around 8.6 nm when ∆T = ±0.1 °C (up 85.8 nm when ∆T = ±1 °C).

In short, the environmental error sources only contribute to the δ_x_, δ_y_ and δ_z_ motion errors. Deviations in the X- and Y-axis are only ≈0.1 nm, when the temperature range increases from ±0.1 °C to ±1 °C. However, the main problem would appear with the high discrepancies obtained in the Z-axis when working at ±1 °C. The variation in that case may be more than 100 nm, due to the change in the evaluated temperature control level. The δ_z_ error motion turns then critical when establishing the requirement for the controlled environment.

### 4.2. Final Error Evaluation

The error budget methodology concludes with the evaluation of the measurement deviations in three axes. That considers the mathematical model (hypothesis and simplifications) and the different error sources. For the studied NanoPla case, the final expression of the error vector ∆P_S_ (∆P_S,x_, ∆P_S,y_, ∆P_S,z_) corresponds with Equation (10). Regarding error sources, the Table 3 summarizes all specific contributions of instrumental, alignment and environmental influences. Then, this subsection presents the final error estimation of the developed 2D-long range stage. The contribution of multiple error sources for every systematic error is assumed as the quadratic sum of the different terms. That is root-sum-of-squares, in view of random and independent influences. Table 4 reviews the obtained errors for ∆T = ±0.1 °C. Nevertheless, two different temperature situations are following analyzed to observe not only the variations in the error motion variables, but also their propagation over the metrology loop.

**Table 4:** Final contribution of the individual considered errors components (∆T = ±0.1 °C): motion, rotation and orthogonality errors.

Geometric Error	Parameter	Estimated Value
**Motion errors** (3)	**δ_x_**	16.14 nm
	**δ_y_**	16.14 nm
	**δ_z_**	19.05 nm
**Rotation errors** (3)	**ε_x_**	1.54 × 10^−8^ rad
	**ε_y_**	1.54 × 10^−8^ rad
	**ε_z_**	2.86 × 10^−7^ rad
**Orthogonality errors** (3)	**α_xy_**	1.09 × 10^−6^ rad
	**α_yz_**	1.14 × 10^−6^ rad
	**α_zx_**	1.31 × 10^−6^ rad

**Figure 9:**
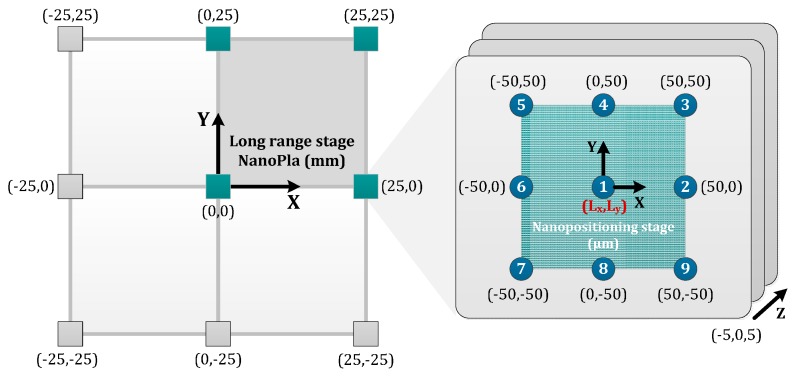
Studied positions in the NanoPla error budget: critical long range stage location (25,0) mm and three-level grid of short range displacements (±50 μm in XY-plane and ±5 μm along Z-axis). (**Left**) XY-long range motion schematization; (**Right**) XYZ-nanostage extreme positions.

The ∆P_S_ error vector depends on the displaced range by the moving platform and the nanopositioner. Hence, it is necessary to analyze several cases that characterize these two long and short range motions, respectively. In view of their higher negative influence, the two shown cases consider the extreme positions of the moving platform travel (50 mm × 50 mm) and the nanostage (100 μm × 100 μm × 10 μm). In particular, and due to the symmetry, the next presented results consider the worst long range location, regarding the final error; *i.e.*, (25,0). At that position, the nanopositioning stage also influences the error. The evaluated short range grid considers nine points at three vertical levels: ±50 μm in XY-plane and ±5 μm along Z-axis. This is illustrated in Figure 9.

**Figure 10:**
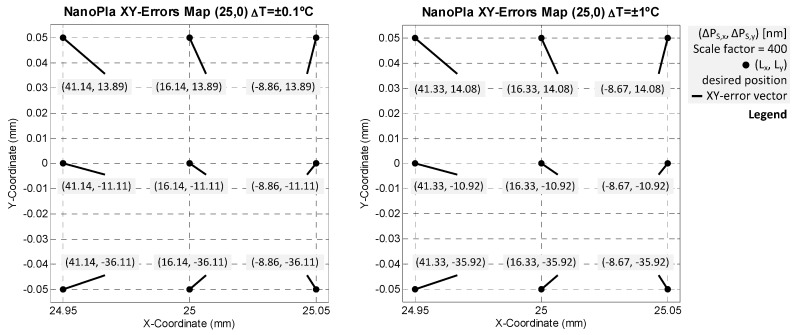
XY-error mapping results (error values in mm) at the (25,0) position, considering different environmental conditions: (**Left**) ∆T = ±0.1 °C; (**Right**) ∆T = ±1 °C.

**Figure 11:**
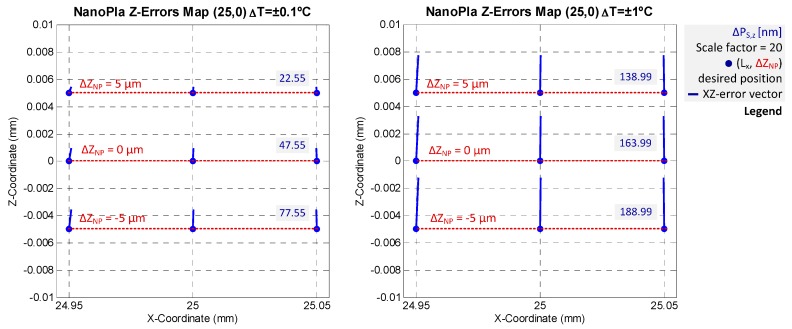
XZ-error mapping results (error values in mm) at the (25,0) position, considering different environmental conditions: (**Left**) ∆T = ±0.1 °C; (**Right**) ∆T = ±1 °C.

The error budget results in the cited positions are presented below. Two different temperature environments are studied: ∆T = ±0.1 °C and ∆T = ±1 °C. Then, Figure 10 and Figure 11 include the XY- an XZ-error mapping and measurement deviations results for both thermal control situations, respectively.

The obtained model and results of the error vector for the different analyzed positions demonstrate that there are more important than other influences. The higher contributions mainly concern the motion errors along the three axes. The Z-error accomplishes a value greater than 160 nm when the temperature is controlled up ±1 °C. In addition, this error vector component is the one that has the largest relative change. XY-discrepancies in both environmental analyzed cases are kept almost exact. The maximum error during positioning is approx. 41 nm in X-axis and 36 nm in Y-axis, though these results contemplate all the error sources and their highest estimated contribution. These extreme situations could not take place during operation. Furthermore, several parameters will be minimized after optimization and corrected by calibration procedures, which would decrease the final error.

The main error influence is a consequence of the temperature control. Then, the complete thermal loop should be known properly. That means to measure accurately the temperature, to understand the dimensional changes that occur in the physical system, and to manage the thermal drift. In view of the studied case, thermal expansion of components supposes elevated changes in the final error budget result, specifically along vertical direction. Thereby, the first proposal for improving the design is based on a better environmental control and a new design of the capacitance probes coupling. The temperature control at sub-micrometer scale is challenging, when dimensional changes should be minimized down to this range. To have a sophisticated environmental control results essential to reduce a high error and uncertainty source. That means, for example, the use of an additional climatic chamber in the metrology lab to protect the place where the nanopositioning stage operates, with a temperature control range of ±0.1 °C.

Other error sources with significant influence have to be mentioned. First of all, the form error of the plane mirrors implies relative high deviations in X- and Y-axis. The global flatness of these reflectors should be characterized, to establish the precise surface topography and to apply a software correction, according to these irregularities. The right parallelism between the capacitance probe and target is also a challenging issue. The difficult task of its measurement supposes a noteworthy contribution, regarding the magnitude order of the NanoPla working features. Thus, this parameter for this first prototype should be carefully analyzed during the assembly and first experimentation with the whole stage. Orthogonality terms are also influenced by the assembly. In order to achieve accuracy during the positioning of the different stage parts, the measurement could be carried out by comparison to precise physical standards of known length or angles (squareness) and uncertainty propagation. Moreover, other measurement alternatives could be taken into account. For example, an autocollimator was studied for squareness characterization with worse result than using the CMM. Calibration parameters for squareness errors of the CMM were better than the combined uncertainty of the autocollimator.

## 5. Conclusions

This work has presented the advantageous use of the error budget methodology to improve the measurement uncertainty during the design of a 2D-long range nanopositioning stage. The study begins with the mathematic modelling of the system metrology loop. For this reason, the developed prototype has been described first, defining its novel three-layer architecture and the two-stage strategy. In order to simplify the studied case, different simplifications and hypothesis are justified. Thus, a simpler expression of the error vector can be obtained to relate the different error sources propagation. One significant assumption that has been demonstrated is the rigid body behavior, in view of the finite element analysis validation of the whole stage.

The analysis has continued with the determination and estimation of the error sources. These contributions have been classified into three groups: instrumental, alignment and environmental influences. Laterly, the vector that represents the difference between the actual and the ideal position for the NanoPla stage has been calculated. The final results of the error budget in terms of measurement deviations demonstrate the existence of some preeminent contributions. Therefore, the metrological performance of the system is improved by focusing efforts on the best technical and economical solutions during the design phase by using the error budget conclusions. Results of this error vector for the NanoPla project show that the preeminent contributions mainly affect the motion errors along the three axes. In particular, critical contributions are the environmental conditions, form error of the plane mirror reflectors and decisive manufacturing and assembly tasks of the metrological loop components. Numerical results with a ∆T = ±0.1 °C temperature control concludes in a maximum error vector of approximately (41,36,48) nm for the X-, Y- and Z-axis, respectively. This agrees with the established operating features at submicrometer scale.

By using the conclusions obtained from the error budget here applied, the results could still be further improved in the future. The global flatness of the plane mirrors surface should be characterized to accurately evaluate their surface irregularities. The right parallelism between the capacitance probe and target should be carefully analyzed during the assembly and first experimentation with the whole stage, such as the characterization of the squareness between all three axes. Finally, once the stage is fully operative, its positioning deviation could also be minimized by applying both parameter optimization algorithms and calibration techniques that would also assure its measurement traceability.
